# Structural basis of lipid-droplet localization of 17-beta-hydroxysteroid dehydrogenase 13

**DOI:** 10.1063/4.0000886

**Published:** 2025-10-27

**Authors:** Shenping Liu, Associate Research Fellow

**Affiliations:** Pfizer, Groton, CT, USA

## Abstract

Hydroxysteroid 17-beta-dehydrogenase 13 (HSD17B13) is a hepatic lipid droplet-associated enzyme that is upregulated in patients with non- alcoholic fatty liver disease. Recently, there have been several reports that predicted loss of function variants in HSD17B13 protect against the progression of steatosis to non-alcoholic steatohepatitis with fibrosis and hepatocellular carcinoma. Here we report crystal structures of full length HSD17B13 in complex with its NAD^+^ cofactor, and with lipid/detergent molecules and small molecule inhibitors from two distinct series in the ligand binding pocket. These structures provide insights into a mechanism for lipid droplet-associated proteins anchoring to membranes as well as a basis for HSD17B13 variants disrupting function. Two series of inhibitors interact with the active site residues and the bound cofactor similarly, yet they occupy different paths leading to the active site. These structures provide ideas for structure-based design of inhibitors that may be used in the treatment of liver disease.

